# C-reactive protein as a predictor of prognosis following curative resection for colorectal liver metastases

**DOI:** 10.1038/sj.bjc.6603558

**Published:** 2007-01-09

**Authors:** V K H Wong, H Z Malik, Z Z R Hamady, A Al-Mukhtar, D Gomez, K R Prasad, G J Toogood, J P A Lodge

**Affiliations:** 1 HPB and Transplant Unit, St. James's University Hospital, Leeds LS9 7TF, UK

**Keywords:** C-reactive protein, colorectal liver metastases, inflammatory response, survival

## Abstract

There is increasing evidence that systemic inflammatory response has a positive correlation with a poorer outcome in patients undergoing resection for solid tumours. The aim of this study was to analyse the impact of an elevated C-reactive protein (CRP), an outcome following curative resection for colorectal liver metastases. One hundred and seventy patients who underwent curative resection for colorectal liver metastases were included in the study. Laboratory measurements of haemoglobin, white cell, platelets, albumin and CRP were taken on the day before surgery. Elevated CRP (>10 mg l^−1^) was present in 54 (31.8%) patients. The median survival of patients with an elevated CRP was 19 months (95% CI 7.5–31.2 months) compared to 42.8 months (95% CI 33.2–52.5 months) for those with a normal CRP, *P*=0.004. Similarly, when assessing disease-free survival, patients with an elevated CRP had poorer disease-free survival (median of 11.8 months (95% CI 6.4–17.3) compared to median of 15.1 months (95% CI 11.1–19.1)), *P*=0.043. The result of the study showed that an elevated preoperative CRP is a predictor of poor outcome in patients undergoing curative resection for colorectal liver metastases.

Colorectal cancer is the third commonest malignancy in UK with around 32 000 people diagnosed with colorectal cancer each year (Cancerstats, 2003; www.cancerresearchuk.org). Of that, around 20–25% of patients will have liver metastases (CRLM) at presentation and a further 40–50% will develop metachronous CRLM following colorectal surgery. Over the last two decades, liver resection has been established as the standard therapy for CRLM and offers the best chance of a potential cure with a 5-year survival of over 40% (Finch *et al*, 2006; Simmonds *et al*, 2006). However, disease recurrence is common with about two-thirds of patients who had liver resection for CRLM developing recurrent disease, and half of these patients had the disease recur in the remnant liver (Steele *et al*, 1991; Nordlinger *et al*, 1994; Fong *et al*, 1997; Hamady *et al*, 2006).

Several studies have looked for possible prognostic factors indicating poor survival outcome and disease recurrence after initial liver resection for CRLM (Fong *et al*, 1999; Seifert *et al*, 2000; Takahashi *et al*, 2003; Tanaka *et al*, 2004; Hamady *et al*, 2006). Primary colorectal adenocarcinoma stage and grade, size, distribution and number of liver metastases, presence of extrahepatic disease, resection margins and lymph nodes status are among the potential prognostic factors but to date, no consensus have been reached.

Recently, there is increasing evidence that inflammation, both local and systemic, has a causal link in the pathogenesis of many solid tumours (Balkwill and Mantovani, 2001; Coussens and Werb, 2002). Infiltration of proinflammatory macrophages, cytokines and chemokines in the tumour microenvironment predispose the tumour to further progression, growth, invasion and metastasis. Gunter *et al* (2006) showed an association between chronic low-grade inflammation, as evidenced by elevated C-reactive protein (CRP), with an increased risk of colorectal cancer. In addition, raised CRP is associated with an increased risk of developing early recurrence and poor outcome following colorectal surgery (McMillan *et al*, 1995, 2003; Canna *et al*, 2005). This suggests that it is not only the intrinsic properties of tumour cells that determine invasion and metastasis, but also the tumour microenvironment.

The aim of this study is to examine the relationship between inflammation, as evidenced by elevated CRP measured before surgery and outcome in patients who had liver resection for CRLM.

## MATERIALS AND METHODS

Patients undergoing resection for colorectal liver metastases had CRP measured preoperatively. CRP (reference range being 10 mg l^−1^ or less) measurements were taken on the day before surgery with none of the patients showing clinical signs of sepsis. The criteria for acceptance for surgery included fitness for major resection and lack of disseminated or irresectable extrahepatic disease identified by computerised tomography (CT) or MRI scan. In all cases, the colorectal primary had been previously resected and the patients had recovered fully from that procedure. Patients who underwent neo-adjuvant therapy were excluded from this study. Intraoperative ultrasound was used as an adjunct to the preoperative radiological investigations. Resection was performed using the Cavi-Pulse Ultrasonic Surgical Aspirator (CUSA, Model 200 T, Valley Lab., Boulder, CO, USA). If necessary, an intermittent Pringle manoeuvre was used with 15 min of ischaemia followed by 5 min of reperfusion.

In accordance with our unit protocol, all patients undergoing liver resection were offered adjuvant therapy in the form of 5-FU/folinic acid, unless they had received adjuvant therapy following their colonic resection within the past one year.

Patients were followed up at specialist clinics, with a minimum follow-up period of 1 year at the time of writing (range 1−5 years; median 28 months). No patients were lost to follow-up. An intensive policy of postoperative surveillance exists within this unit. Patients have three monthly chest and abdominal CT performed during the first postoperative year, then 6 monthly during year 2. From year 3 to 5, a CT scan is performed yearly and finally at year 7 and year 10 of follow-up. Tumour markers carcinoembryonic antigen ((CEA), CA19-9) and liver function tests are performed during each clinic visit. The data examined included patient demographics; liver resection histology; prehepatectomy CEA and CA19-9 tumour marker; prehepatectomy CRP; postoperative morbidity/mortality results as well as recurrence and survival figures.

## STATISTICS

An SPSS version 9 statistical programme was used to analyse the data. The Student's *t*-test and χ^2^ tests were used to analyse differences among groups of patients with high or normal CRP. Where variables did not follow a normal distribution, the Mann–Whitney test was applied. Kaplan–Meier survival curves were used to analyse patient outcome. Patients who died in the postoperative period were excluded from the analysis of outcome. A Cox regression analysis was then performed in a step-wise manner in order to perform a multivariable analysis of clinico-pathological factors that impact both overall and disease-free survival.

## RESULTS

A total of 170 patients were included in this study. Of these patients, 106 (62%) were males and 64 (38%) females. The mean age of patient at time of surgery was 64 years (range 37–87 years; s.d. 9.86 years). A total of 86 (50.6%) patients had synchronous disease. All patients underwent liver resection. There were 76 (44.7%) patients who underwent an anatomical resection, a further 45 (26.5%) patients underwent a combination of anatomical and nonanatomical resection whereas the remaining 49 (28.8%) patients underwent a nonanatomical resection. A total of 57% of patients had a ‘major’ (three or more Couinaud segments) resection performed. The in-hospital mortality rate was 3% and 29 (17%) patients had postoperative complications.

Preoperative CRP was elevated (>10 mg l^−1^) in 54 (31.8%) patients. The differences in the clinico-pathological features of patients with a normal compared to an elevated CRP are presented in Table 1. Of note is that patients with an elevated CRP had no significant differences in the ‘T’ or ‘N’ stage of the primary tumour. However, patients with an elevated CRP tended to have larger metastases as well as increased CA19-9 levels compared to those with normal CRP.

### Outcome

**Figure 1:**
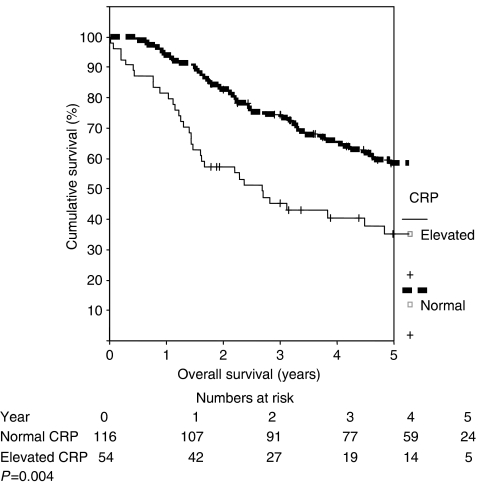
Overall survival stratified according to CRP.

**Figure 2:**
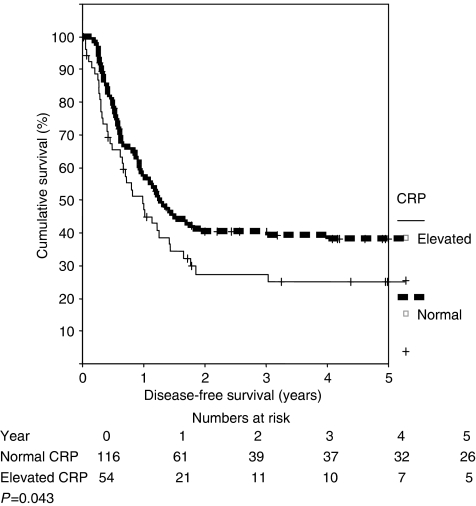
Disease-free survival stratified according to CRP.

Overall there were 80 (47%) patients who died during the time period of this study. Of these, there were six non-cancer-related deaths. Thus, the cancer-specific survival was 56%. The median survival of patients with an elevated CRP was 19 months (95% CI 7.5–31.2 months) compared to 42.8 months (95% CI 33.2–52.5 months) for those with a normal CRP, *P*=0.004 (Figure 1). Similarly, when assessing disease-free survival, patients with an elevated CRP had poorer disease-free survival (median of 11.8 months (95% CI 6.4–17.3) compared to median of 15.1 months (95% CI 11.1–19.1)), *P*=0.043 (Figure 2). The results of univariate and multivariable analysis of the predictors of overall survival are shown in Tables 2 and 3, respectively. Of note is the fact that an elevated CRP was the only predictor of poor survival on univariate analysis. However on multivariable analysis, an elevated CRP as well as primary ‘T’ stage both predicted for poorer overall survival. When the multivariable analysis was repeated for disease-free survival, only the presence of an elevated CRP predicted for poorer outcome, *P*=0.011 (HR 4.07; 95% CI 1.36–12.19).

## DISCUSSION

Tumour progression is a complex process that depends not only on the intrinsic properties of the tumour, but also on its interaction with the host cells. Although the link between inflammation and cancer was first proposed by Virchow in 1863, it is only in last two decades that we are beginning to delineate and have an understanding of this intricate network of interactions.

This study showed that elevated preoperative CRP present in almost one-third of patients with CRLM and is associated with a poor overall as well as disease-free survival in patients who had liver resection for colorectal metastases. This is consistent with other studies that found a positive correlation between elevated CRP concentrations, before surgery and a poor outcome in patients who had curative primary tumour resection (Nozoe *et al*, 2001; McMillan *et al*, 2003; Canna *et al*, 2005; Crumley *et al*, 2006). However, this is the first study to identify such a relationship among patients who have undergone curative resection for CRLM. This finding has important implications for selection of patients for surgery and the presence of an elevated CRP should be considered when counselling patients upon the likelihood of a ‘curative resection’.

We also found that elevated CRP was associated with presence of larger metastases size and elevated CA19-9. It is possible that any impact upon survival of an elevated CRP may be as a result of this association. However, as neither of these factors were independent predictors of survival on multivariable analysis, it appears that the elevated CRP is the main factor contributing to the poorer outcome among these patients. Interestingly, there was no association between ‘T’ and ‘N’ characteristics of the primary and an elevation in CRP.

The basis for the relationship between elevated CRP and poor prognosis is unclear and there are several possible explanations. Elevated CRP level may simply reflect a nonspecific inflammatory response to tumour necrosis or local tissue damage. Alternatively, it may be indicative of a favourable environment for the establishment and growth of distant metastases. Serum level of vascular endothelial growth factor (VEGF), an angiogenic factor, is increased in the presence of raised CRP concentration (Xavier *et al*, 2006). Angiogenesis plays an important role in tumour growth and is associated with a poor outcome in patients with GI tumours (Tanigawa *et al*, 1997; Fondevila *et al*, 2004). In addition, interleukin (IL) -1 and -6 network is upregulated (Vidal-Vanaclocha *et al*, 2000; Miki *et al*, 2004). Interleukin 1 is involved in the development of metastasis in animal studies and IL-6, apart from being a growth factor, promotes resistance to apoptosis (Vidal-Vanaclocha *et al*, 1994, 2000; Chauhan *et al*, 1997; Frassanito *et al*, 2001; Jee *et al*, 2001). This creates a microenvironment that favours tumour angiogenesis, proliferation, growth and metastases.

Host immune response plays a role in the evolution of tumour growth. An impaired host immune response, evidenced by a weak lymphocyte infiltration at tumour margin, is associated with a poor prognosis in patients who had liver resection for CRLM (Okano *et al*, 2003). There is an inverse relationship between CRP levels and tumour lymphocyte infiltration, with a raised CRP concentration indicative of a weak infiltration of lymphocytes at the periphery of the tumour (Canna *et al*, 2005). The significance of systemic inflammatory response in the pathogenesis of cancer has important clinical implications.

Several novel therapies targeting the inflammatory pathways are in clinical trial (Balkwill and Mantovani, 2001). Tumour necrosis factor (TNF) antagonists, chemokine and IL-6 antagonism, nonsteroidal anti-inflammatory agents and statin drugs are among the potential therapies. Selective Cox-2 inhibitors were of particular interest with promising initial results but adverse cardiovascular risks have limited their usage (Mukherjee *et al*, 2001; Thun *et al*, 2002; Yau *et al*, 2003).

In summary, the results of this study showed that elevated CRP levels, measured before surgery, are present in a significant proportion of patients with CRLM. This is found to predict for a poor survival outcome in patients who had liver resection performed.

## Figures and Tables

**Table 1:** Clinico-pathological features

**Factor**	**Normal CRP (10 mg l^−1^ or less) (*N*=116)**	**Elevated CRP (>10 mg l^−1^) (*N*=54)**	**Significance**
Age (mean)	63.7	64.7	0.577
Synchronous disease	56	30	0.502
Male gender	73	33	0.802
Multiple (4 or more) metastases	27	12	0.568
			
*Primary nodal status*			
N0	32	10	0.371
N1	51	27	
N2	9	6	
			
*Primary T stage*			
T1	1	3	0.111
T2	8	3	
T3	57	19	
T4	24	15	
			
CEA	61.5	63	0.820
CA19-9	53.9	70.9	0.013
Large metastases size (> 50 mm)	34	31	0.003
Positive margin	23	15	0.641

CEA=carcinoembryonic antigen; CRP=C-reactive protein. Mann–Whitney *U*-test.

**Table 2:** Univariate analysis of overall survival

**Factor**	***P*-value**
Older age (years)	0.536
Gender (males)	0.522
CEA	0.353
CA19-9	0.838
Large (>50 mm)	0.661
metastases size	
Positive resection	0.461
Margin	
Synchronous	0.309
disease	
Primary tumour	0.754
nodal positivity	
Poorer tumour	0.124
primary T-stage	
Multiple (4 or more)	0.244
Metastases	
Poor clinical risk score	0.972
Elevated CRP	<0.001

CEA=carcinoembryonic antigen; CRP=C-reactive protein. MSKCC clinical risk score – poor scores defined as scores 3–5 (Fong *et al*, 1999).

**Table 3:** Multivariable analysis of overall survival

**Factor**	**Hazard ratio (95% confidence interval)**		***P*-value**
Older age (years)	1.04	1.00–1.09	0.051
Gender (males)	1.09	0.51–2.32	0.816
CEA	0.99	0.99–1.00	0.785
CA19-9	0.99	0.99–1.00	0.766
Large (>50 mm)	0.99	0.97–1.01	0.827
metastases size			
Positive resection	0.56	0.11–2.86	0.488
Margin			
Synchronous	0.92	0.43–1.95	0.836
disease			
Primary tumour	0.60	0.30–1.19	0.149
nodal positivity			
Poorer tumour	1.82	1.10–3.00	0.019
primary T-stage			
Multiple (4 or more)	2.06	0.91–4.66	0.785
Metastases			
Poor clinical risk	0.707	0.21–2.61	0.747
score			
Elevated CRP	2.86	1.09–7.49	0.032

CEA=carcinoembryonic antigen; CRP=C-reactive protein. MSKCC clinical risk score– poor scores defined as scores 3–5 (Fong *et al*, 1999).

## References

[bib1] Balkwill F, Mantovani A (2001) Inflammation and cancer: back to Virchow? Lancet 357: 539–545 11229684 10.1016/S0140-6736(00)04046-0

[bib2] Cancer Research UK Information Centre (2003) CancerStats. http//:info.cancerresearchuk.org/cancerstats

[bib3] Canna K, McArdle PA, McMillan DC, McNicol A-M, Smith GW, McKee RF, McArdle CS (2005) The relationship between tumour T-lymphocyte infiltration, the systemic inflammatory response and survival in patients undergoing curative resection for colorectal cancer. Br J Cancer 92: 651–654 15700032 10.1038/sj.bjc.6602419 PMC2361875

[bib4] Chauhan D, Kharbanda S, Ogata A, Urashima M, Teoh G, Robertson M, Kufe DW, Anderson KC (1997) Interleukin-6 inhibits Fas-induced apoptosis and stress-activated protein kinase activation in multiple myeloma cells. Blood 89(1): 227–234 8978296

[bib5] Coussens LM, Werb Z (2002) Inflammation and cancer. Nature 420: 860–867 12490959 10.1038/nature01322 PMC2803035

[bib6] Crumley ABC, McMillan DC, McKernan M, Going JJ, Shearer CJ, Stuart RC (2006) An elevated C-reactive protein concentration, prior to surgery, predicts poor cancer-specific survival in patients undergoing resection for gastro-oesophageal cancer. Br J Cancer 94: 1568–1571 16685271 10.1038/sj.bjc.6603150 PMC2361311

[bib7] Finch RJB, Malik HZ, Hamady ZZR, Al-Mukhtar A, Adair R, Prasad KR, Lodge JPA, Toogood GJ (2006) Outcome following liver resection for colorectal liver metastases: effect of type of resection. Br J Surg (in press) 10.1002/bjs.5640 17657718

[bib8] Fondevila C, Metges JP, Fuster J, Grau JJ, Palacin A, Castells A, Volant A, Pera M (2004) p53 and VEGF expression are independent predictors of tumour recurrence and survival following curative resection of gastric cancer. Br J Cancer 90: 206–215 14710231 10.1038/sj.bjc.6601455 PMC2395306

[bib9] Fong Y, Cohen AM, Fortner JG, Enker WE, Turnbull AD, Coit DG, Marrero AM, Prasad M, Blumgart LH, Brennan MF (1997) Liver resection for colorectal metastases. J Clin Oncol 15: 938–946 9060531 10.1200/JCO.1997.15.3.938

[bib10] Fong Y, Fortner J, Sun RL, Brennan MF, Blumgart LH (1999) Clinical score for predicting recurrence after hepatic resection for metastatic colorectal cancer: analysis of 1001 consecutive cases. Ann Surg 230: 309–318 10493478 10.1097/00000658-199909000-00004 PMC1420876

[bib11] Frassanito MA, Cusmai A, Iodice G, Dammaco F (2001) Autocrine interleukin-6 production and highly malignant multiple myeloma: Relation with resistance to drug-induce apoptosis. Blood 97: 483–489 11154226 10.1182/blood.v97.2.483

[bib12] Gunter MJ, Stolzenberg-Solomon R, Cross AJ, Leitzmann MF, Weinstein S, Wood RJ, Virtamo J, Taylor PR, Albanes D, Sinha R (2006) A prospective study of serum C-reactive protein and colorectal cancer risk in men. Cancer Res 66: 2483–2487 16489056 10.1158/0008-5472.CAN-05-3631

[bib13] Hamady ZZR, Cameron IC, Wyatt J, Prasad RK, Toogood GJ, Lodge JPA (2006) Resection margin in patients undergoing hepatectomy for colorectal liver metastasis: A critical appraisal of the 1 cm rule. Eur J Surg Onco 32: 557–563 10.1016/j.ejso.2006.02.001 16580811

[bib14] Jee SH, Shen SC, Chiu HC, Tsai WL, Kuo M (2001) Overexpression of interleukin-6 in human basal cell carcinoma cell lines increases anti-apoptotic activity and tumorigenic potency. Oncogene 20: 198–208 11313947 10.1038/sj.onc.1204076

[bib15] McMillan DC, Canna K, McArdle CS (2003) Systemic inflammatory response predicts survival following curative resection of colorectal cancer. Br J Surg 90: 215–219 12555298 10.1002/bjs.4038

[bib16] McMillan DC, Wotherspoon HA, Fearon KC, Sturgeon C, Cooke TG, McArdle CS (1995) A prospective study of tumor recurrence and the acute-phase response after apparently curative colorectal cancer surgery. Am J Surg 170: 319–322 7573721 10.1016/s0002-9610(99)80296-7

[bib17] Miki C, Konishi N, Ojima E, Hatada T, Inous Y, Kusunoki M (2004) C-reactive protein as a prognostic variable that reflects uncontrolled up-regulation of the IL-1–IL-6 network system in colorectal carcinoma. Dig Dis Sci 49: 970–976 15309885 10.1023/b:ddas.0000034556.48527.6e

[bib18] Mukherjee D, Nissen SE, Topol EJ (2001) Risk of cardiovascular events associated with selective COX-2 inhibitors. JAMA 286: 954–959 11509060 10.1001/jama.286.8.954

[bib19] Nordlinger B, Vaillant JC, Guiguet M, Balladur P, Paris F, Bachellier P, Jaeck D (1994) Survival benefit of repeat liver resections for recurrent colorectal metastases: 143 cases. Association Francaise de Chirurgie. J Clin Oncol 12: 1491–1496 8021741 10.1200/JCO.1994.12.7.1491

[bib20] Nozoe T, Saeki H, Sugimachi K (2001) Significance of preoperative elevation of serum C-reactive protein as an indicator of prognosis in esophageal carcinoma. Am J Surg 182: 197–201 11574097 10.1016/s0002-9610(01)00684-5

[bib21] Okano K, Maeba T, Modoguchi A, Ishimura K, Karasawa Y, Izuishi K, Goda F, Usuki H, Wakabayashi H, Maeta H (2003) Lymphocytic infiltration surrounding liver metastases from colorectal cancer. J Surg Oncol 82(1): 28–33 12501166 10.1002/jso.10188

[bib22] Seifert JK, Bottger TC, Weigel TF, Gonner U, Junginger T (2000) Prognostic factors following liver resection for hepatic metastases from colorectal cancer. Hepato-Gastroenterology 47: 239–246 10690615

[bib23] Simmonds PC, Primrose JN, Colquitt JL, Garden OJ, Poston GJ, Rees M (2006) Surgical resection of hepatic metastases from colorectal cancer: a systematic review of published studies. Br J Canc 94: 982–999 10.1038/sj.bjc.6603033 PMC2361241 16538219

[bib24] Steele Jr G, Bleday R, Mayer RJ, Lindblad A, Petrelli N, Weaver D (1991) A prospective evaluation of hepatic resection for colorectal carcinoma metastases to the liver: Gastointestinal Tumor Study Group Protocol 6584. J Clin Oncol 9: 1105–1112 2045852 10.1200/JCO.1991.9.7.1105

[bib25] Takahashi S, Inoue K, Konishi M, Nakagouri T, Kinoshita T (2003) Prognostic factors for poor survival after repeat hepatectomy in patients with colorectal liver metastases. Surgery 133: 627–634 12796730 10.1067/msy.2003.151

[bib26] Tanaka K, Shimada H, Fujii Y, Endo I, Sekido H, Togo S, Ike H (2004) Pre-hepatectomy prognostic staging to determine treatment strategy for colorectal cancer metastases to the liver. Arch Surg 389: 371–379 10.1007/s00423-004-0490-y 15605168

[bib27] Tanigawa N, Amaya N, Matsumura M, Shimomatsuya T (1997) Correlation between expression of vascular endothelial growth factor and tumor vascularity, and patient outcome in human gastric carcinoma. J Clin Oncol 15: 826–832 9053510 10.1200/JCO.1997.15.2.826

[bib28] Thun MJ, Henley SJ, Patrono C (2002) Nonsteroidal anti-inflammatory drugs as anticancer agents: mechanistic, pharmacologic, and clinical issues. J Natl Cancer Inst 94: 252–266 11854387 10.1093/jnci/94.4.252

[bib29] Vidal-Vanaclocha F, Amezaga C, Asumendi A, Kaplanski G, Dinarello CA (1994) Interleukin 1 receptor blockade reduces the number and size of murine B16 melanoma hepatic metastases. Cancer Res 54: 2667–2672 8168095

[bib30] Vidal-Vanaclocha F, Fantuzzi G, Mendoza L, Fuentes AM, Anasagasti MJ, Martin J, Carrascal T, Walsh P, Reznikov LL, Kim SH, Novick D, Rubinstein M, Dinarello CA (2000) IL-18 regulates IL-1b-dependent hepatic melanoma metastasis via vascular cell adhesion molecule-1. Proc Natl Acad Sci USA 97: 734–739 10639148 10.1073/pnas.97.2.734 PMC15399

[bib31] Xavier P, Belo L, Beires J, Rebelo I, Martinez-de-Oliveira J, Lunet N, Barros H (2006) Serum levels of VEGF and TNF-a and their association with C-reactive protein in patients with endometriosis. Arch Gynecol Obstet 273: 227–231 16208475 10.1007/s00404-005-0080-4

[bib32] Yau M, Kargman S, Lam EC, Kelly CR, Zheng Y, Luk P, Kwong E, Evans JF, Wolfe MM (2003) Inhibition of cycooxygenase-2 by rofecoxib attenuates the growth and metastatic potential of colorectal carcinoma in mice. Cancer Res 63: 586–592 12566300

